# Evaluating the Prototype of a Clinical Decision Support System in Primary Care: Qualitative Study

**DOI:** 10.2196/69875

**Published:** 2025-08-20

**Authors:** Susanne M Köhler, Svea Holtz, Michaela C Neff, Jannik Schaaf, Michael von Wagner, Beate S Müller, Dania Schütze

**Affiliations:** 1 Institute of General Practice, Goethe University Frankfurt, Theodor-Stern-Kai 7, Frankfurt am Main, 60590, Germany, 49 696301 ext 7267; 2 Institute of Medical Informatics, Goethe University Frankfurt, University Medicine Frankfurt, Frankfurt am Main, Germany; 3 Department of Medical Information Systems and Digitalization, University Medicine Frankfurt, Frankfurt am Main, Germany; 4 Institute of General Practice, University of Cologne, Faculty of Medicine and University Hospital Cologne, Cologne, Germany

**Keywords:** clinical decision support systems, primary care, user-centered design, usability testing, qualitative research

## Abstract

**Background:**

General practitioners are confronted with a wide variety of diseases and sometimes diagnostic uncertainty. Clinical decision support systems could be valuable to improve diagnosis, but existing tools are not adapted to the requirements and workflow in the primary setting. In project SATURN (Smart physician portal for patients with unclear disease), the prototype of a clinical decision support system based on artificial intelligence is being developed together with users specifically for primary care in Germany. It aims to reduce diagnostic uncertainty in cases of unclear and rare diseases and focuses on 3 medical fields. A user-centered design approach is applied for prototype development and evaluation.

**Objective:**

This study evaluates the usability of a high-fidelity prototype and explores aspects of user experience like the subjective impression, satisfaction, and areas of improvement.

**Methods:**

A total of 5 general practitioners participated in the evaluation, which consisted of (1) a remote think-aloud test, (2) a postsession interview, and (3) a survey with the System Usability Scale. During the think-aloud tests, the participants verbalized their thoughts and actions and solved several vignette-based tasks. Remarkable observations were logged, transcribed with quotes, and analyzed for usability problems and positive findings. All observations and interview responses were deductively assigned to the following categories: (1) content, (2) comprehensibility, (3) user-friendliness, (4) layout, (5) feedback, and (6) navigation. Usability problems were described in detail and solutions for improvement proposed. Median and total scores were calculated for all questionnaire items.

**Results:**

The evaluation detected both strengths and areas for improvement. The participants particularly liked the clear and well-structured layout of the prototype. Key issues identified were content-related limitations, such as the inability to enter unlisted symptoms, medications, and examination findings. Also, participants found the terminology for laboratory not suitable to their needs. Another key issue was a lack of user-friendliness concerning the time required to input medication plans and lab values. Participants expressed a need for faster data entry, potentially through direct imports from practice management systems or laboratory files. Adding symptom duration, weighting symptoms, and incorporating hereditary factors were suggestions made for improvement. Overall, the SATURN prototype was deemed useful and promising for future clinical use, despite the need for further refinements, particularly in the areas of data entry, as this is a key obstacle to its use.

**Conclusions:**

The usability evaluation methods combined proved to be location independent and easy to use. They provided important findings on usability issues and improvements that will be implemented in a second high-fidelity prototype, which will also be tested by users. Technically demanding user requirements, such as direct data transfer from the practice management system and entry options that require complex data models, were beyond the scope of this project, but should be considered in future development projects.

## Introduction  

General practitioners (GPs) are confronted with a wide variety of diseases and sometimes with diagnostic uncertainty [1]. As a large number of rare diseases are not commonplace in primary care, it is impossible for GPs to know all these symptoms and make differential diagnoses based purely on their experience and knowledge. Recognizing patterns in symptom complexes does not work in this case [2]. This can be very burdensome for patients, as the diagnosis of a rare disease can often take years. During this time, misdiagnosis and numerous consultations are the rule, and sometimes the diagnosis nevertheless remains unclear [3-5]. For GPs, clinical decision support systems (CDSS) could be especially valuable to improve diagnosis of frequent diseases with unusual manifestations of symptoms or rare diseases. However, “Google Search” or regular symptom checkers are not apt for the medical workflow, and specific tools for this setting are lacking. According to a scoping review of Harada et al [6], screening and diagnosis of chronic diseases are the main targets of CDSS in a primary care setting, but uncommon chronic diseases are not covered. A mixed-methods systematic review evaluating CDSS providing recommendations to primary care professionals by Meunier et al [7] including studies from 1998 to 2021 revealed that most systems were developed and used in the United States. Diagnosis was only presented in 7/45 of the investigated CDSS and only 1 system aiming at iatrogenic prevention was developed for the German market.

Rare diseases—defined in the European Union as diseases with a prevalence of not more than 5 affected persons per 10,000 [8]—are especially challenging in terms of diagnosis. Diagnosis support is supplied by specific databases for rare diseases (eg, “Orphanet”) and 3 types of specific search engines: most of them are based on the analysis or comparison of genetic and phenotypic data (eg, “Phenomizer”), some on information retrieval (eg, “FindZebra”), and few on machine learning. Often, they lack direct data integration and do not provide information on when the system was last updated. Not all are fully developed systems or publicly available. Furthermore, these tools are not adapted to the requirements, needs, and workflow of GPs and are not designed together with users [9].

In general, when successfully designing eHealth apps, the iterative involvement of potential users at different stages of the development process is necessary, which is the basis of a user-centered design process [10-12]. To evaluate prototypes, repeated usability testing is crucial. Various behavioral and technology acceptance models describe that factors such as ease of use are an important factor for adoption and actual use [13,14]. Quantitative methods, especially standardized questionnaires like the System Usability Scale (SUS), which give an overall measure of usability, are most frequently used, often alone, and sometimes in combination with qualitative instruments like task completion, think-aloud (TA), interviews, heuristic testing, and focus groups [15]. While effectively depicting general user acceptance and facilitating comparisons with other systems, standardized questionnaires may fall short in identifying the specifics and extent of inherent usability problems, which can be captured by the application of qualitative methods. However, the exact process of qualitative usability testing, analyzing, and the reporting in literature is rare [15].

In the “Smart physician portal for patients with unclear disease” (SATURN) project, funded by the German Federal Ministry of Health, an artificial intelligence (AI)-based CDSS is being developed with and exclusively for GPs to aid in diagnosing unclear and rare diseases. The proof-of-concept study focuses on 3 medical fields (gastroenterology, pneumology, and endocrinology) and provides decision support based on medical guidelines and clinical cases from university hospitals. These fields were selected because the university hospitals involved in the project have clinical expertise in these areas and the quality of the available data was deemed suitable for the planned measures.

A total of 3 different AI methods are applied, to our knowledge for the first time in a CDSS for unclear diagnosis: diagnosis support based on (1) rule-based systems, (2) case-based reasoning, and (3) machine learning. In addition, when a rare disease is detected, further assistance is offered in terms of direct connections to specific databases delivering disease-specific knowledge and support concerning specialized care centers and patient organizations [16].

In a previous study [17], we described the requirements analysis as the first stage of the user-centered design process of the CDSS SATURN, which iteratively involves a group of 5 GPs. Initially, user requirements were identified through workshops and interviews, and a task model was developed in the follow-up. Based on the requirements analysis and subsequent prioritizing regarding impact and technical feasibility, first mockups and then a first high-fidelity, clickable prototype of the portal’s user interface were designed [18]. The latter was initially tested through usability inspections and refined [19].

In this publication, we report the evaluation of the first high-fidelity SATURN prototype. The objective of the study was to investigate usability problems of the CDSS (eg, in terms of data entry, comprehensibility, layout, etc) at an early stage, as well as positive findings, advantageous system solutions that should not be changed. Also, we wanted to determine the GPs’ satisfaction with the CDSS and improvement requests. Furthermore, we put special emphasis on our method mix applied for usability testing and on the process of data analysis and reporting which is not well documented in literature.

## Methods       

### Design          

We used a primarily qualitative design to evaluate the first high-fidelity, clickable prototype of the SATURN portal with a small group of GPs. The AI modules, which will later provide the results for decision support, were not yet connected in this version. For the usability test, sample data for diagnostic suggestions was, therefore, stored in the system.

The study consisted of three parts: (1) a remote TA test to identify usability issues (problems and positive findings), (2) a postsession interview, and (3) an SUS questionnaire to evaluate user satisfaction and find out improvement requests. All 3 parts were carried out consecutively in individual remote sessions. During the test, the GPs had to perform tasks and were advised to “think aloud,” that is, to utter all thoughts, for example, what they were looking for, what they were expecting, or what was irritating them. This approach was chosen to understand the requirements, objectives, reasons, impressions, and sensations behind the users’ observed behavior [20,21]. The researchers who conducted and analyzed the tests (SMK, SH, and DS) were not part of the development team. The study was conducted and reported in accordance with the Consolidated Criteria for Reporting Qualitative Research (COREQ) [22].

### Setting and Sampling        

A group of 5 GPs, male and female, was invited. All group members took part in the usability testing between July and August 2023. The participants were recruited by email using purposeful sampling [23], all were interested in digitalization topics and had already been involved in the requirements analysis. They received written information on the study and provided written informed consent. All tests were conducted remotely in individual online sessions via Zoom (Zoom Communication). The SATURN prototype was accessible via a URL and an assigned user and password and could be used in the web browser. All sessions were audio- and videotaped.

### Ethical Considerations

The Ethics Committee of the Goethe University Frankfurt am Main approved the SATURN study (case 2022‐1088). The Ethics Committee waived the need for a separate ethics vote on the inquiry of GPs (case 2022‐629). All participants received written information on the study and provided a signed informed consent. All data were pseudonymized before analysis. Participants received €150 (average conversion rate in August 2023: US $1 = €0.92) for taking part in the usability test.

### Data Collection

#### SATURN Prototype

**Figure 1.**
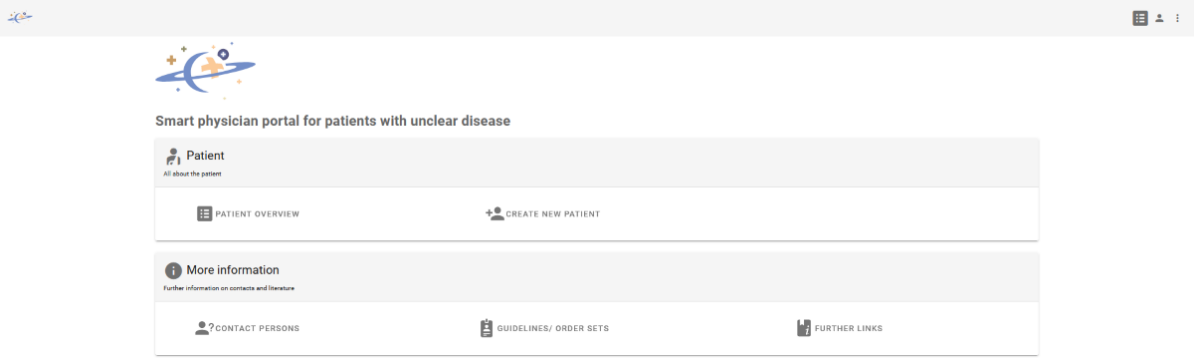
Screenshot: Start page.

SATURN consists of a start page (see Figure 1) with the following options: (1) Patient and (2) More information.

With the “Patient” option, logged-in GPs get an overview of all patients (Patient overview) and enter new patient data to receive diagnostic suggestions (Create new patient). There are seven steps for data entry under “Create new patient” for individual data categories (basic data, diagnoses, symptoms, medication, laboratory and vital values, and examinations) followed by an overview page of all entries (summary). From this data entry page, the GP can both access the page with possible AI diagnosis suggestions (results) and the case closure page where the GP can enter the final diagnosis once it has been confirmed.

With the “More information” option, GPs get more information on selected diseases and contact options (More information). In addition to contacts to specialists and support groups (Contacts), they will also find links to selected guidelines (Guidelines and Order sets) and websites (Further links).

A navigation bar on top leads through the data entry pages. Screenshots of all major SATURN pages are provided in Multimedia Appendix 1 (all screenshots are translated from German into English for this publication).

#### Remote Think-Aloud Test

The interdisciplinary study team consisted of researchers with backgrounds in sociology, medicine, medical sciences, and medical informatics, all of whom had experience with qualitative data collection. DS, SH, and SMK developed a test plan with test objectives, an interview guide, and a case vignette with 5 tasks to fulfill [10,20]. An internal pilot test was performed. All tests were moderated by SH.

The case vignette was based on a case report of Graves disease (an autoimmune thyroid disease) by Sharma [24]. The case report was selected as it depicted one of the SATURN disease patterns (endocrinology) and took place in an outpatient setting. It was shortened (parts about treatment and further course of the disease were removed) and information on medication dosages was added. It was narratively adapted to the primary care setting in Germany and checked for plausibility by an internist specializing in endocrinology. The following tasks, which were based on the task model developed in the requirements analysis [17], had to be performed: (1) entering patient data from the case vignette, (2) determining the most plausible diagnosis proposal, (3) returning to the start page, (4) concluding the case, and (5) finding information on patient organizations and care centers for rare diseases in the system.

Before the video conference, the participants received an email providing an access link and the case vignette with tasks (see Multimedia Appendix 2) but were advised not to study it before the test. During the remote test, the GPs shared their screen while performing the tasks so that the process could be observed by the moderator and audio- and video-recorded (a written record was created later). The test persons verbalized their actions and what was going through their mind while using the system, for example, expectations, comprehension problems, or questions [11,20,25]. If necessary, the moderator supported the think-aloud process with questions like "What are you thinking?", "What are you looking at right now?", "What are you doing at the moment?", or "What would you be doing if I wasn’t here"?

#### Postsession Interview

The postsession interview took place immediately after the usability test to evaluate the GPs’ subjective impression of the CDSS and their desire for improvement (satisfaction and user experience). A total of three open-ended questions were asked: (1) What did you like about the system?, (2) What should we improve?, and (3) What is your overall impression: What do you think about the system?

#### System Usability Scale

The evaluation of the portal’s usability was completed by applying the standardized SUS questionnaire, developed by Brooke [26]. The questionnaire was filled out online by the test persons right after the postsession interview. The SUS contains 10 items in the form of closed questions or statements on the assessment and learnability of software systems [27]. A total of 5 statements are formulated positively (eg, “I thought the system was easy to use”), 5 negatively (eg, “I found the system unnecessarily complex”). Agreement with the statements is measured using a 5-point Likert scale including verbal descriptions of the endpoints (1=strongly disagree and 5=strongly agree). We used the German version of the questionnaire [28].

### Data Analysis

#### Remote Think-Aloud (TA) Test

##### Categories for Usability Issues

To evaluate the video recordings, a member of the study team (SH or SMK) created a protocol using Microsoft Excel 2016. Observations pointing out usability issues when carrying out the tasks were described, associated with quotes, and a time stamp was noted. The minute-takers checked each other’s protocols for clarity and completeness. The observations were categorized into usability problems and positive findings. First, all detected usability problems were compiled in a table and sorted by location (On which system page did the problem occur?) and by similarity (Did several test persons experience the same problem?). Second, similar to the categories of Li et al [29] and Gorshid et al [30], who also evaluated user interfaces through TA-tests, we defined categories that depicted relevant aspects of the system’s usability: (1) Content, (2) Comprehensibility, (3) User-friendliness, (4) Layout, (5) Feedback, and (6) Navigation (see Table 1). All findings were deductively assigned to these categories. In the course of the analysis, which was conducted by SH and SMK and supervised by DS, subcategories were inductively defined for each category to further classify the usability issues on a descriptive level. The subcategories were created in a discursive process between the researchers. Positive usability findings were summarized separately in an Excel file and sorted accordingly.

**Table 1.** Definition of categories for usability issues.

Category	Explanation of usability problem(s)
Content	The CDSS^a^ texts are medically inaccurate or inappropriate or the terminology used is not understood by the test persons.
Comprehensibility	The meaning of texts, instructions, and the purpose of CDSS components cannot be quickly grasped by the test persons.
User-friendliness	The test persons are unable to handle the CDSS easily and use it with minimal effort (in terms of time, clicks, and mental energy).
Layout	The test persons are unable to recognize relevant information on the screen or they overlook it. They describe aesthetic problems and point out redundant information.
Feedback	The feedback the test persons receive from the user interface is not relevant or does not meet the users’ expectations. A lack of feedback is criticized.
Navigation	The test persons express problems navigating the system.

a CDSS: clinical decision support system.

##### Description of Usability Problems

Finally, a detailed summary of each usability problem with the following information was created: (1) Title, (2) Severity, (3) Location, (4) Category, (5) Subcategory, (6) Description (partly with screenshot), (7) Number of test persons experiencing the problem, (8) Possible cause, and (9) Recommendation. A classification of 5 severity levels was used, based on the traditional Nielsen scale and its further development [31,32] (see Table 2). The severity level was determined in the team (SMK, SH, DS, and MCN), and the recommendations were developed by MCN.

**Table 2.** Definition of severity levels for usability problems.

Gradation	Severity level	Definition
Idea	0	The user makes a suggestion for an additional function.
Minor usability problem	1	The usability problem leads to temporary irritation of the user. The task can be completed.
Moderate usability problem	2	The usability problem leads to moderate irritation for the user. The task can be completed via detours or with a delay. Some users fail.
Critical usability problem	3	The usability problem causes considerable irritation or comprehension problems. The task cannot be completed.
Bug	4	Software error.

##### Postsession Interview

All responses were transcribed with quotes and time stamps, and analyzed according to structuring content analysis [33]. Quotes were assigned to the same categories and subcategories that were used for the usability issues. This made it possible to look at the results of the TA test and the interviews in conjunction.

##### System Usability Scale

With a large number of test persons, an overall score can be calculated from the SUS answers, representing the system’s overall usability numerically [34]. Since the number of test subjects (n=5) in our study is not sufficient for a quantitative statement based on the overall score [35], we evaluated the median, total, and range per item. The aim was to identify possible outlier items and to triangulate the results with those from the usability tests and the postsession interviews.

## Results

### Participant and Interview Characteristics

The characteristics of the 5 participating GPs and the duration of the usability tests are presented in Table 3.

**Table 3.** Participant and interview characteristics (n=5).

Characteristic	Participant ID				
	1	2	3	4	5
Age (years)	44	45	34	50	36
Sex	Male	Female	Male	Male	Female
Additional qualifications or special focus	Emergency care, focus on geriatrics and palliative care.	Intensive and emergency care.	No additional qualifications.	Diabetologist, nephrologist, focus on hypertensiology and nutritional medicine.	Focus on rare diseases.
Time working as GP^a^ (years)	10	7	2	9	3.5
Employment status	Self-employed	Employed	Employed	Self-employed	Employed
Duration of usability test and interviews (min:s)	55:50	56:00	47:47	37:15	42:50

a GP: general practitioner.

### Remote Think-Aloud (TA Test) and Postsession Interview

Our results are presented below based on the defined categories (see Table 1). Individual findings are reported by way of example and supported by quotes. In addition to usability problems in the narrower sense (severity levels 1‐3), the results include problems designated as “ideas,” that is, missing functions that are not yet provided in the system (severity level 0). Also, positive usability findings are reported, that is, aspects that were praised by the participants and should be retained.

Table 4 provides an overview and brief description of all usability problems in the narrower sense (severity levels 1‐3), including their frequency. Problem details can be found in Multimedia Appendix 3. Bugs (severity level 4) that occurred during the test were reported to the developers immediately after the sessions, fixed, and are not discussed further in this paper.

**Table 4.** Usability problems by category and subcategory.

Category	Subcategory	Usability problem	Persons (out of 5) who experienced the problem, n (%)
		Brief description	
Content	Lab values input	Unusual naming of stored laboratory values.	5 (100)
		Inconsistent preset options for laboratory values.	1 (20)
		Contents of “Small blood count” and “Large blood count” not clear.	1 (20)
	Symptoms input	Input of symptoms not already stored in system not possible.	4 (80)
	Diagnoses input	Input of confirmed diagnosis only possible with *ICD^a^* code.	4 (80)
	Medication input	Input of medications not already stored in system not possible.	2 (40)
		Input of the dose for drug combinations unclear.	1 (20)
		“Night” medication administration time missing.	1 (20)
	Entering findings	Input of instrumental examination findings not already stored in system not possible.	1 (20)
	Entering vital signs	Imprecise labeling of blood pressure input field.	1 (20)
	*ICD* code display	Incomplete display of the *ICD* text.	1 (20)
Comprehensibility	Button label	Label of “Insert lab value set” button incomprehensible.	5 (100)
		“Positive/Negative” label not suitable for laboratory results.	2 (40)
		Label of “Back to overview” button imprecise.	1 (20)
		Label of “Go to case input” button incomprehensible.	1 (20)
		Label of “Contact persons” button not clear.	1 (20)
	Input field	Lack of instructions for input.	4 (80)
		Unclear function of the “Diagnostic history” input field.	1 (20)
	Icons	Unclear function of the Info icon.	1 (20)
		Inappropriate icons for diagnosis input.	1 (20)
	Explanatory text	Contradictory explanatory texts for diagnosis input.	1 (20)
User-friendliness	Time required	Excessive time required to input medication plan.	3 (60)
		Excessive time required to input lab values.	1 (20)
	Input support	Large number of stored units.	2 (40)
		Complicated input of confirmed diagnoses.	1 (20)
	Operation	Lack of export or print function.	1 (20)
		Lack of filter functions.	1 (20)
Layout	Clarity	Poor localization of the laboratory value input field.	2 (40)
		Poor localization of the “Contact persons” button.	1 (20)
		Poor localization of the results list of patient organizations.	2 (40)
		Visibility of the findings text lacking.	1 (20)
	Input field	Poor recognition of the symptom input field.	1 (20)
	Structure	Awkward prioritization of medication preselection.	1 (20)
Feedback	Saving	Lack of confirmation when saving symptoms.	2 (40)
		Lack of confirmation when saving the confirmed diagnosis.	1 (20)
	Feedback lacking	Closed cases marking is lacking.	2 (40)
Navigation	Page navigation without any bar	Direct navigation back to home page not visible.	2 (40)
	Page navigation with bar	Nonadaptive labeling of the navigation bar.	1 (20)

a *ICD*: *International Classification of Diseases*.

Table 5 depicts a summary of all positive usability findings.

**Table 5.** Positive usability findings by category and subcategory.

Category	Subcategory	Brief description positive findings	Persons (out of 5) who mentioned the issue, n (%)
User-friendliness	Input support	Input support for diagnoses (*ICD*^a^ code).	2 (40)
		Input support for symptoms (symptom list).	2 (40)
		Input support for medications (letters combination).	1 (20)
	Operation	Text can be transferred from other files.	1 (20)
Content	Lab values input	Input of capital letters possible.	1 (20)
	Probabilities	Diagnosis suggestions with indication of probability.	1 (20)
Layout	Clarity	Clear page design (start page and contact persons).	2 (40)
Navigation	Page navigation with bar	Quick movement through the pages possible.	1 (20)
	Further links	Links with contact details available.	1 (20)
Comprehensibility	Input field	Input field for notes available (Symptoms page).	1 (20)

a *ICD*: *International Classification of Diseases*.

### Content

Many problems were of a content-related nature, including some that meant that a task could not be completed correctly. For example, it proved to be problematic that the participants did not have a suitable means of entering symptoms, medication, and examination findings that were not listed in the drop-down menu. Some used the open text field “Notes” for this purpose, but entries in this field are not taken into account in the diagnosis search.

I can just write “daytime tiredness” and “nightmares,” and “weight loss over weeks” in the comments [refers to the open text field “Notes”]. To give a time frame, I would add that too, as well as “gastrointestinal symptoms for about 4 weeks.”

[GP 2, Usability problem 7]

When entering the laboratory values, some of the terminology used for the stored laboratory values was not appropriate. All test persons tried to search for laboratory values under common abbreviations (eg, TSH and GFR) but did not find them.

Now I’ll try again with the TSH [thyroid stimulation hormone]. Maybe I have to scroll there - at least that’s how it was with the others too. No, nothing comes up. No, it doesn’t seem to exist.

[GP1, Usability problem 18]

The presentation of the results with probabilities, which corresponded to the expectations of the test subjects, was positively emphasized.

Yes, that’s good with the probability indication here in percentage, I know it from Ada Health too, yes, great.

[GP 1, Positive finding]

During the usability tests, the GPs also made some suggestions as to how the content of the CDSS could possibly be improved (ideas). For example, it was suggested that the duration of symptoms should be requested and that the weighting of symptoms should be made possible. It was also suggested that the important information “rhythmic/arrhythmic” should be added to the heart rate and that physical examination findings should be entered (eg, enlarged liver or spleen). The interviews also emphasized the importance of family history in making a diagnosis.

What would not be bad either would be a sub-item on previous family illnesses…especially with regard to heredity, genetic predisposition - something like that would be very important [GP mentions diabetes, coronary heart disease] … that is definitely part of it.

[GP 2, Interview]

### Comprehensibility

The usability test made it clear that some of the labeling and functions of buttons, input fields, and symbols were imprecise or incomprehensible. All test subjects had problems understanding the name and function of the “Insert lab value set” button, which was intended to support the input of the large and small blood count.

Can I click on here, somehow...it’s not at this point...it’s not quite clear to me why it’s called something like a laboratory value set, but then there’s only the option of large and small blood count. And if I want something else, I have to enter it at the bottom here. I don’t quite understand that at this point, but it seems to be intentional, so I probably click on this here now.

[GP 1, Usability problem 23]

Some participants were unclear about the function of the “Diagnostic history” input field and the meaning of some symbols. A total of 4 people did not understand what should be entered on the “Case closure” page. Therefore, the corresponding task could not be completed.

Where am I now? Diagnostic accuracy. I’m not quite sure what I’m supposed to do at this point. Should I enter the final diagnosis? Or the presumptive diagnosis it churned out for me?

[GP 1, Usability problem 31]

### User-Friendliness

The test persons rated the time required to enter the medication plan and laboratory values as not user-friendly. The large number of selectable laboratory value units also irritated users and delayed entry.

[GP 5 enters medication with dosage, form of administration, and unit.] And here too, now I’m already really annoyed that I somehow […]. Again, it’s all too detailed for me. It would take far too long in the consultation for me.

[GP5, Usability problem 9]

During the interviews, 3 GPs again discussed the time-consuming entry of medication, laboratory values, and diagnoses. In this context, the desire for direct import from the practice management system was expressed several times.

I found it difficult to enter medication and laboratory values... and then having to enter the standard value myself, that kills it a bit’...or the best would be to import it directly from the system.

[GP 3, Interview]

To further improve user-friendliness, a test person suggested that it should be possible to mark diagnoses that have already been excluded in the results list.

Of course it would be nice for me if I could now exclude things directly… [gives an example ]. if I could cross them out directly or something.

[GP 3, Idea 5]

The principle of input support through stored diagnoses, symptoms, and medications used on several pages appealed to the participants and was rated as user-friendly.

Ok, you also entered the ICD code. That’s good.

[GP 5, Positive findings]

### Layout

The GPs generally liked the clear, well-structured layout of the portal, with 4 people praising it in the interviews.

Relatively‚ “clean,” ie, clear, not so many incomprehensible things, kept simple and easy to start, largely self-explanatory.

[GP 3, Interview]

However, a lack of clarity was observed regarding details on 2 pages. For example, 2 test subjects had to search for the field for entering new laboratory values. On the “Contact persons” page, only clinic contacts were directly recognizable on the screen. The list of patient organizations below was only accessible by scrolling and was not found by 2 test persons.

### Feedback

The test persons missed provision of feedback from the system when saving the symptoms and the confirmed diagnosis. The fact that whether or not a case had already been closed was not recognizable in the patient overview led to considerable irritation.

And then I go to “Close case”...Okay. Then I go again here to “Close case,” because I would have expected it to say that the case is closed… but… so I would have thought that now there would be a check mark, or it says “Case closed”, but well, okay.

[GP 2, Usability problem 34]

### Navigation

All GPs found the navigation bar at the top of the screen, which led them through the various pages of the SATURN portal, very easy to use. In the interviews, the navigation bar was praised several times.

The navigation in the portal is a great solution... that I can jump directly to these individual items with master data and symptoms in this bar at the top... I think that’s very good.

[GP 5, Interview]

### Overall Rating

Overall, the final personal assessment in the interviews was positive for all test persons, and the portal was rated as a very useful, helpful, and interesting diagnosis support tool and as a not unlikely option for the future. With few exceptions, the GPs succeeded in completing the tasks.

I think it’s still very much in its infancy... I’m really excited to see what comes out of it in the end. I’m still convinced that it makes total sense and that it can still be really good.

[GP 2, Interview]

The subjectively perceived usability of the SATURN prototype was also positive in many respects (see Table 6). Using the SUS questionnaire, the median of the positively formulated items was 4 and 5 respectively (best rating: 5), and the median of the negatively formulated items was 2 and 1 respectively (best rating: 1). Overall, the highest approval was given to items concerning the ease of use and the speed with which the system can be learnt. Correspondingly, items stating the need for technical support and the necessity to learn a lot before getting started met with no approval. This corresponds to the findings of the usability test and interviews, especially the praise of the well-structured layout, the user-friendly input support, and the easy and intuitive navigation. No outlier items were identified, but some test persons found that there was too much inconsistency in the system.

**Table 6.** Usability rating by means of System Usability Scale (SUS) items (n=5).

Item	Median (minimum-maximum)	Summed rating of all participants
Positively formulated items		
I thought the system was easy to use.	5 (3-5)	23
I would imagine that most people would learn to use this system very quickly.	5 (3-5)	23
I found the various functions in this system were well integrated.	5 (3-5)	22
I think that I would like to use this system frequently.	4 (3-5)	20
I felt very confident using the system.	4 (3-5)	20
Negatively formulated items		
I thought there was too much inconsistency in this system.	2 (1-4)	11
I found the system unnecessarily complex.	1 (1-4)	8
I found the system very cumbersome to use.	1 (1-3)	8
I think that I would need the support of a technical person to be able to use this system.	1 (1-3)	7
I needed to learn a lot of things before I could get going with this system.	1 (1-1)	5

## Discussion      

### Principal Findings

The evaluation highlighted strengths, usability problems, and areas for improvement. Key issues included content limitations, such as the inability to enter unlisted symptoms and medications, as well as unfamiliar terminology for laboratory values. In addition, data entry for medication and lab values was seen as cumbersome, with a need for faster input methods. Despite these challenges, participants appreciated the user-friendly navigation and clear layout, considering the prototype promising for future clinical use, though refinements in data entry are needed.

The remote setting allowed location-independent and natural environment testing for GPs, simulating a realistic use case of the portal. The use of a case vignette with tasks also contributed to this. The TA method was quickly adapted and well-accepted by the GPs. It provided us with detailed feedback on already well-implemented user requirements, for example, the clear and concise structure and the simple, intuitive operation of the interface. It also helped us identify the extent and precise causes of the usability issues that were found.

The basic structure of the system was evaluated positively, and the problems encountered seem in line with the status of the prototype (eg, imprecise or incomprehensive labels, buttons or symbols, lack of feedback, etc). For further development, it was important to obtain feedback on the usability of the user interface at this stage. At the same time, dealing with some problems and suggestions is not easy. For example, aspects such as the desire for simple, straightforward entry on the one hand and the need for particularly detailed data collection on the other hand have to be reconciled. During the usability tests, the GPs made some suggestions as to how the content of the CDSS could possibly be improved (ideas). For example, they suggested that the duration of symptoms should be requested, that the weighting of symptoms should be enabled and that hereditary factors should be considered. Although these factors may be relevant for diagnosis, such data would require substantial amendments on the portal’s database (university hospital data and elements), as well as complex algorithms and data models [36,37], which could not be met in our proof-of-concept project. However, such factors would be valuable and should be considered in the future.

It proved to be problematic that the participants did not have a suitable means of entering symptoms, medication, and examination findings that were not listed in the drop-down menu. Although the introduction and design of additional open-entry fields are easily performed, the potential input must also be mapped in the data model to contribute to diagnosis.

The test persons rated the time required to enter the medication plan and laboratory values as not user-friendly. The large number of selectable laboratory value units, which is due to different terminology standards being used (research vs practice and laboratory standard), also irritated users and delayed entry. However, the user requirement for quick data entry is, according to our test results, likely to be a crucial factor for the acceptance of CDSS in general practice settings. Increased workload also proved to be the greatest barrier to using CDSS in primary care in the systematic review by Meunier et al [7]. In this study, CDSS slowness was also addressed as an important technological barrier, next to missing user-friendliness and a lack of integration in the electronic health record (although 28/45 CDSS included in this study were already partially or fully integrated in the electronic health record). Another major organizational barrier identified in this study, which corresponds to our results, was the disruption of the usual workflow. The automated direct transfer of data from practice management systems or from the laboratory files remains a technical challenge for future development projects. This applies especially to countries like Germany where a wide variety of management systems and laboratory standards are being used [38,39]. Here, individual solutions for data transfer need to be developed for each system [40]. To address this issue, a workshop will be held later in the project with representatives from practice management software companies and other experts to discuss possibilities for interoperability standards for practice management systems in Germany. The results will be reported elsewhere.

Overall, the evaluation of the SATURN prototype shows that a system has been created that can be easily integrated into the workflow of GPs and meets the requirements of the participants to a high degree. Such a system offers good prerequisites for being used in everyday practice diagnosis searches and for contributing to faster and better diagnosis finding. At the current early stage of development, however, it is not yet possible to make any statements about the effectiveness and benefits in everyday practice.

The prototype of SATURN is now being further developed, and the usability issues and suggestions are being technically implemented. The next version of the prototype will be tested with 10 GPs, 5 of whom are not yet familiar with the CDSS. The respective evaluation will also include the presentation of AI-based diagnostic suggestions.

### Strengths and Limitations

The combination of the TA usability test, the postsession interview, and the standardized questionnaire provided a comprehensive overall picture of the portal’s usability. Classifying the findings into categories during the analysis enabled us to systematize and triangulate results. The detailed problem descriptions served as the basis for finding appropriate problem solutions and further system development.

A limitation of testing the usability of the SATURN prototype in a preliminary stage of development is that the AI modules were not yet working, so the test results had to be simulated. Therefore, it was not possible to fully evaluate the functionality and the perceived usefulness and acceptance of the system.

Another limitation might be due to the limited sample size in combination with the continuous involvement of the 5 GPs in the development process. For qualitative usability testing, this group size is common and recurring problems can be sufficiently recognized [10,20,41]. When selecting the participants, we made sure to create a diverse sample in terms of gender, age, professional experience, and specialization. During the development process, numerous ideas developed by the GPs were gradually implemented. This could have caused a bias in the subsequent portal evaluation. For this reason, additional GPs will be included in the next evaluation.

### Conclusions

By combining usability tests, postsession interviews, and a standardized questionnaire, a high-fidelity CDSS prototype for GPs was efficiently evaluated with potential users in terms of usability and user satisfaction. The qualitative tools applied were location-independent and easy to use. Solutions for the usability problems detected could be developed and will be implemented in the next prototype. However, the implementation of some technically demanding user requirements, such as direct data transfer from the practice management system and entry options that require complex data models, was beyond the scope of this project. They should be considered in future development projects. In the next iteration, the AI modules for generating results will be integrated, and the presentation of the results will be tested. In addition to usability, the second test will also focus on the system’s acceptability and potential benefits for clinical practice.

## Supplementary material

10.2196/69875 Multimedia Appendix 1 Screenshots of SATURN (smart physician portal for patients with unclear disease) pages.

10.2196/69875 Multimedia Appendix 2 Case vignette and tasks.

10.2196/69875 Multimedia Appendix 3 Details of usability problems.

## Abbreviations

AI: artificial intelligence
CDSS: clinical decision support system
COREQ: Consolidated Criteria for Reporting Qualitative Research
GFR: glomerular filtration rate
GP: general practitioner
SATURN: smart physician portal for patients with unclear disease
SUS: System Usability Scale
TA: think-aloud
