# Supplementary material for: Evaluating the Prototype of a Clinical Decision Support System in Primary Care: Qualitative Study
Source: JMIR Form Res. 2025 Aug 20;9:e69875. doi: 10.2196/69875 (PMC12367354; doi:10.2196/69875)

Screenshot 1: Start page

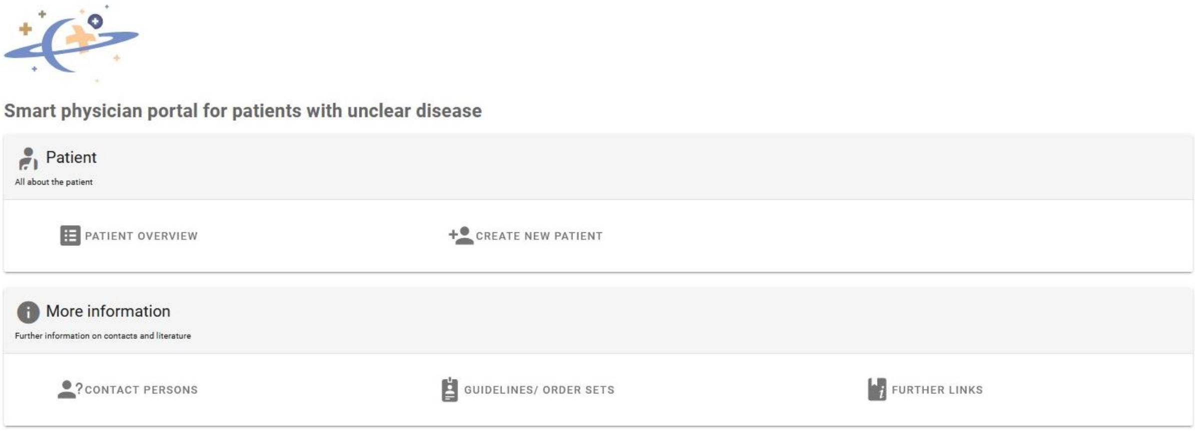

Screenshot 2: Patient overview

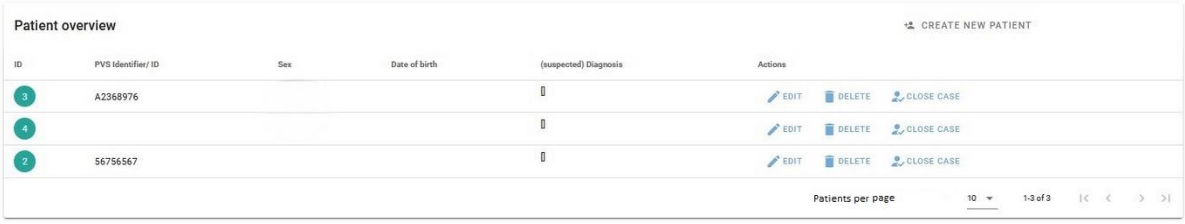

Screenshot 3: Entry of basic data

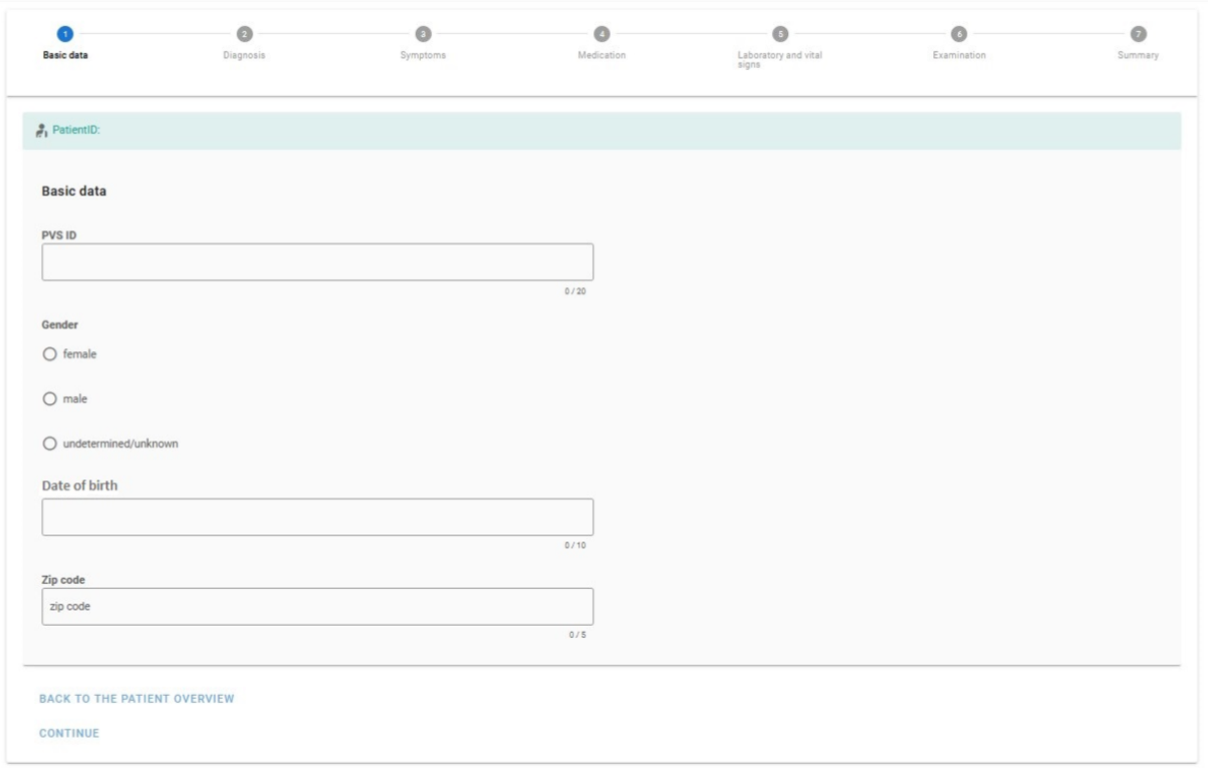

Screenshot 4: Entry of diagnosis data

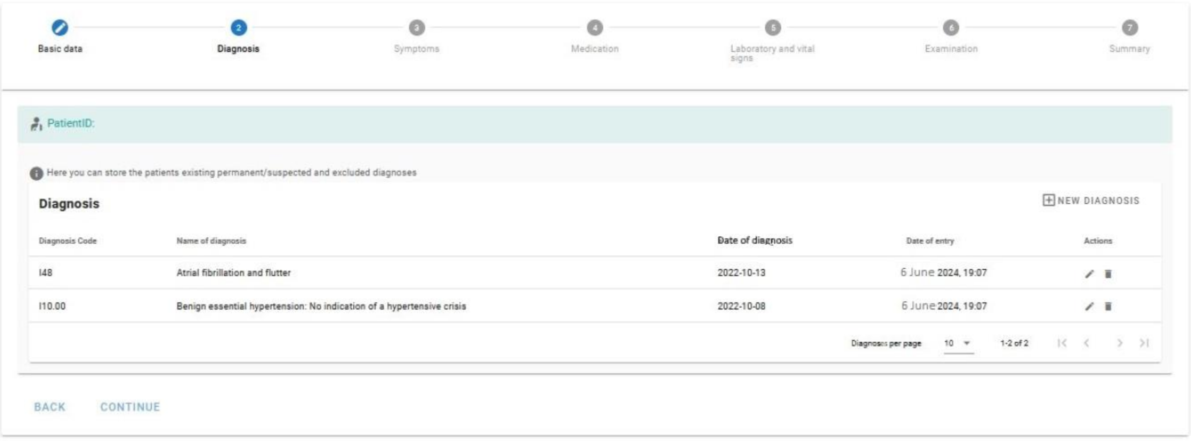

Screenshot 5: Entry of symptoms data

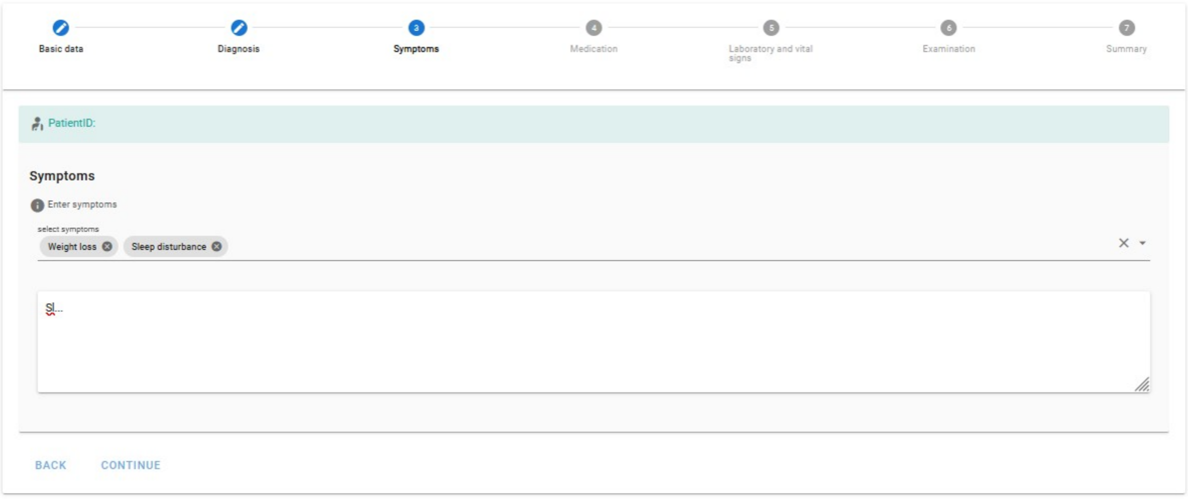

Screenshot 6: Entry of medication data

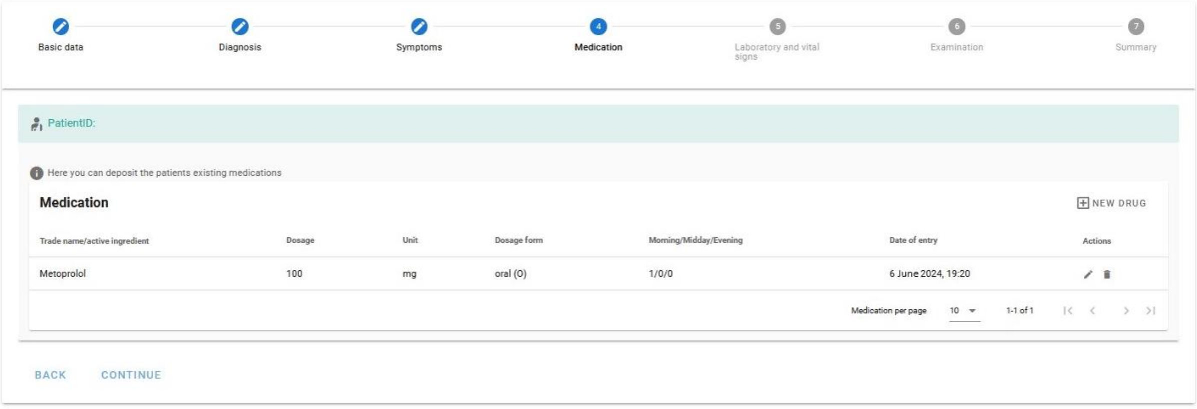

Screenshot 7: Entry of laboratory and vital signs data

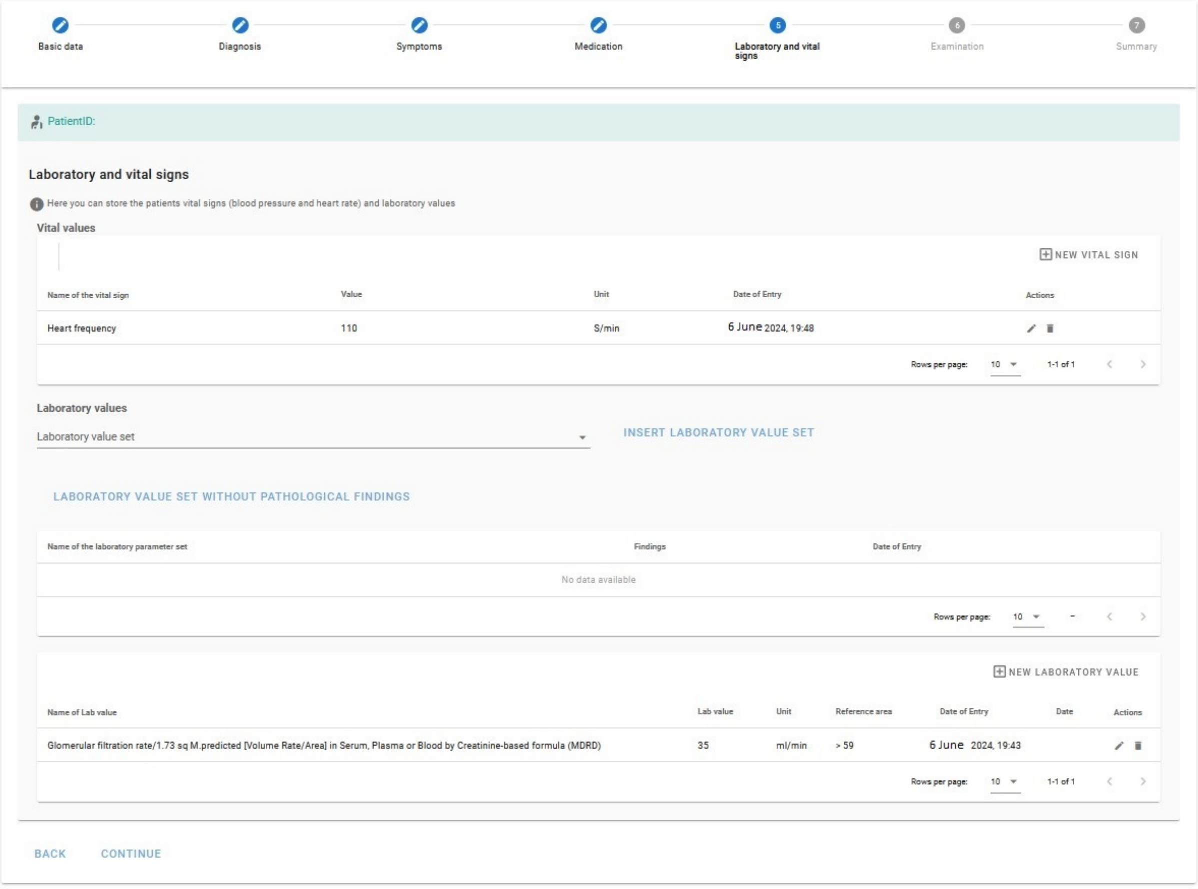

Screenshot 8: Entry of examination data

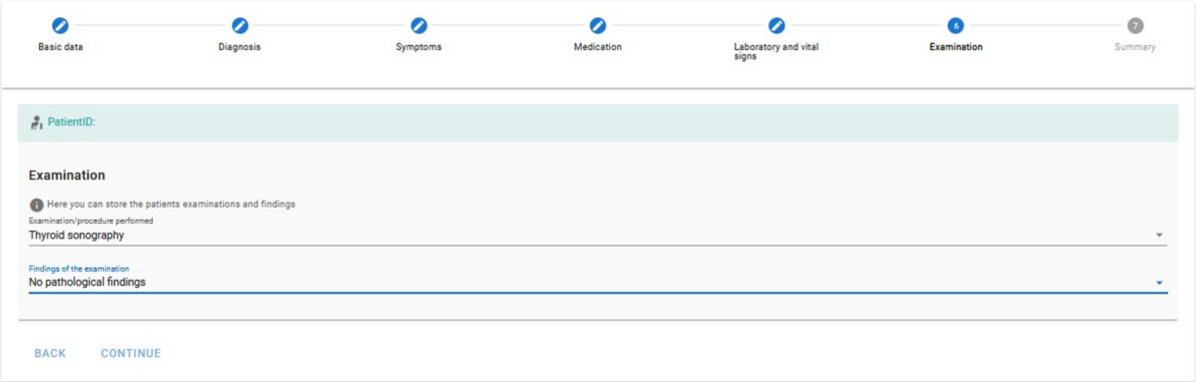

Screenshot 9: Summary

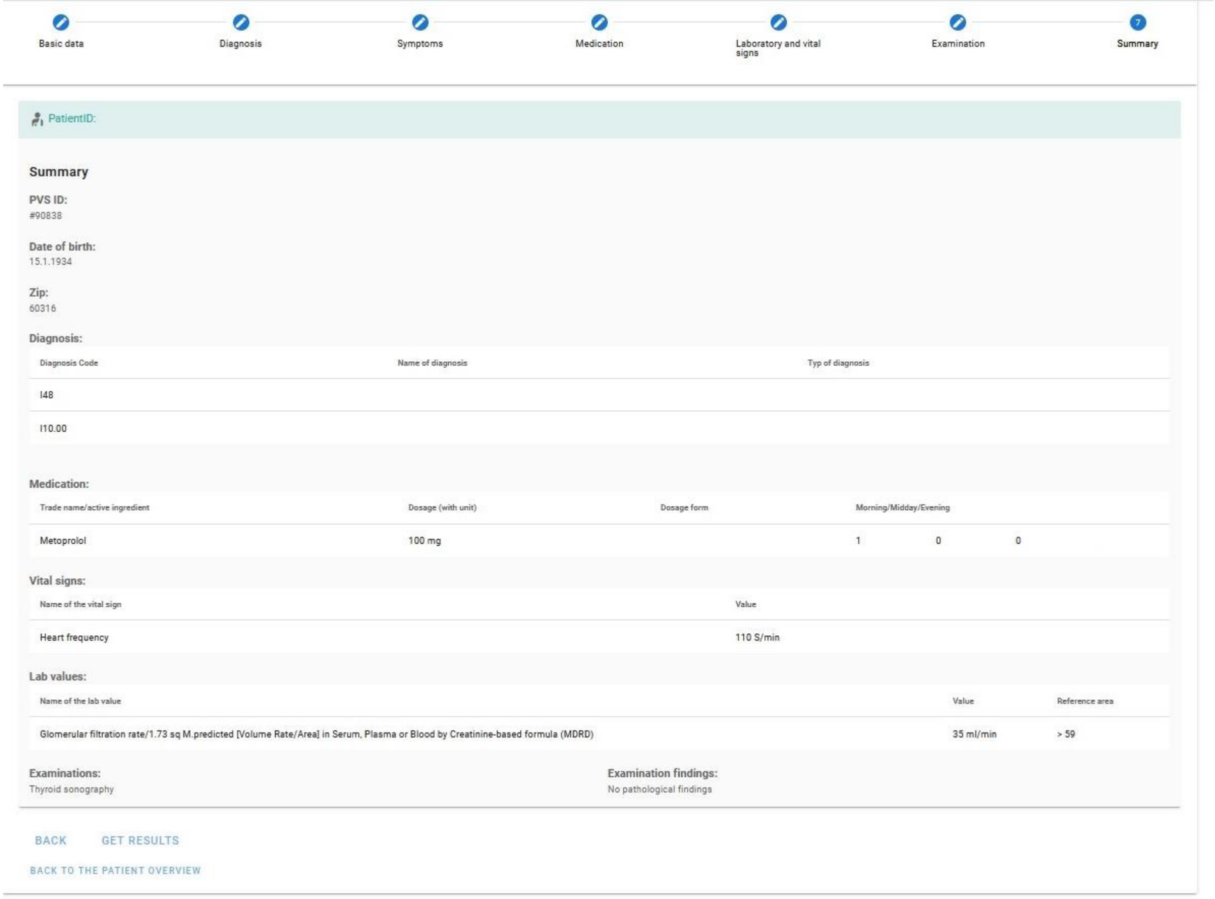

Screenshot 10: Results

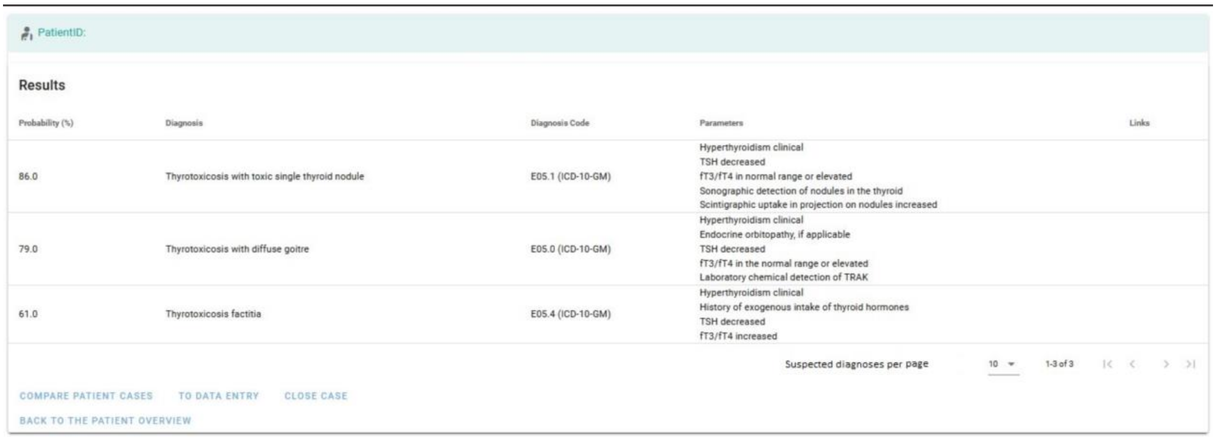

Screenshot 11: Case closure

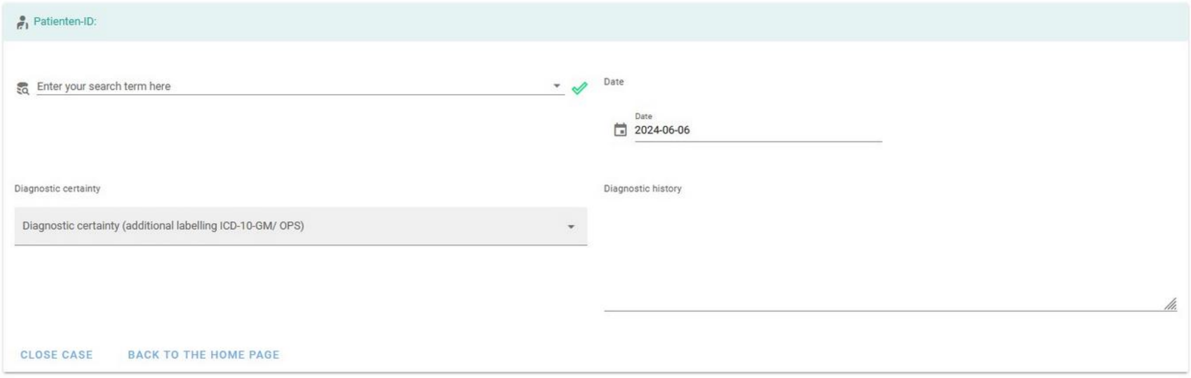

Screenshot 12: Contacts

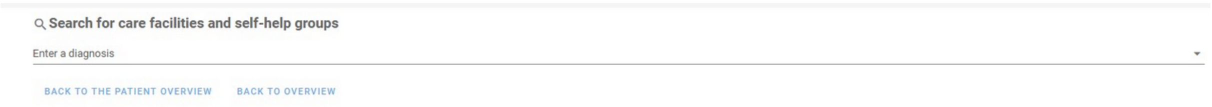

Supplement: Multimedia Appendix 1 [file formative-v9-e69875-s001.pdf]
