# Supplementary material for: Evaluating the Prototype of a Clinical Decision Support System in Primary Care: Qualitative Study
Source: JMIR Form Res. 2025 Aug 20;9:e69875. doi: 10.2196/69875 (PMC12367354; doi:10.2196/69875)
Supplement: Multimedia Appendix 2 [file formative-v9-e69875-s002.pdf]

Mr. Gerd Wismüller, 89 years old, comes to your office for consultation. He reports nausea, vomiting and recurrent diarrhea for a month. He also suffers from loss of appetite and has lost several kilos of weight in recent weeks. He sleeps badly at night and has had repeated nightmares. Also, he often falls asleep in his armchair during the day.

The following data is stored in your patient management system (PMS or PVS in German):

<table>
<tr><th>Category</th><th colspan="5">Information</th></tr>
<tr><td>PMS ID</td><td colspan="5">#90838</td></tr>
<tr><td>Date of birth</td><td colspan="5">15.03.1934</td></tr>
<tr><td>Place of residence</td><td colspan="5">60316 Frankfurt</td></tr>
<tr><td>Confirmed diagnoses/pre-existing conditions</td><td colspan="5">I48.0 Paroxysmal atrial fibrillation
I10.00 Benign essential hypertension: without mention of hypertensive urgency
E78.0 Pure hypercholesterolemia</td></tr>
<tr><td rowspan="7">Medication plan</td><td>Active ingredient</td><td>Dose</td><td>Morning</td><td>Midday</td><td>Evening</td></tr>
<tr><td>Metoprolol ext. release</td><td>100mg</td><td>1</td><td>0</td><td>0</td></tr>
<tr><td>Lisinopril</td><td>20mg</td><td>1</td><td>0</td><td>0</td></tr>
<tr><td>Amlodipine</td><td>5mg</td><td>1</td><td>0</td><td>0</td></tr>
<tr><td>Torasemide</td><td>10mg</td><td>1</td><td>0</td><td>0</td></tr>
<tr><td>Simvastatin</td><td>40mg</td><td>0</td><td>0</td><td>1</td></tr>
<tr><td>Apixaban</td><td>2.5mg</td><td>1</td><td>0</td><td>1</td></tr>
</table>

You make the following findings:

Abdomen soft, bowel sounds brisk, blood pressure 140/90mmHg, tachycardia (HR 100-110/min), arrhythmic, bilateral tremor of hands, thyroid gland not enlarged palpable.

They take blood samples. The results are as follows:

Parameters	Current value	Standard value
Complete blood count with extended testing	Without pathological findings	
Thyroid-stimulating hormone (TSH)	0.006 µU/ml ↓	0.45-4.5 µU/ml
Glomerular filtration rate (GFR)	35 ml/min/1.73 m2 ↓	>59 ml/min/1.73 m2
Creatinine	1.92 mg/dl ↑	0.76-1.27 mg/dl

1. Enter the information relevant to you into the system and find out which diagnosis is most likely based on the data available to you.

2. Then go back to the SATURN portal home page.

You notice Mr. Wismüller's low TSH level. You call your lab and report other values:

**Parameters**	**Current value**	**Standard value**
Free T4	1.57 ng/dl	0.82-1.77 ng/dl

3. Enter this information in the system too.

In this case, you refer your patient to a specialist for further diagnosis of latent hyperthyroidism and therapy. The patient comes back to your office some time later and brings the doctor's letter with them. The diagnosis of Graves' disease has been confirmed.

4. Close the case in the system.

You have another patient with sarcoidosis. She comes to your office today and tells you that she is moving to Leipzig. She is also interested in sarcoidosis self-help groups.

5. Use the system to find out which center in Leipzig could provide your patient with further care for sarcoidosis.

6. Use the system to find information about support groups for people with sarcoidosis.
