# Supplementary material for: Evaluating the Prototype of a Clinical Decision Support System in Primary Care: Qualitative Study
Source: JMIR Form Res. 2025 Aug 20;9:e69875. doi: 10.2196/69875 (PMC12367354; doi:10.2196/69875)
Supplement: Multimedia Appendix 3 [file formative-v9-e69875-s003.pdf]

Usability problems (severity level 1-3) and ideas (severity level 0)

**Usability problem 1:** Poor localization of the ‘Contact persons’ button	
Severity level	1
Location of the problem	Start page
Category	Layout
Subcategory	Clarity
Description	The test person does not see how to navigate to the search for contact persons.
Number of participants	1
Possible cause	On a small screen (laptop and smaller), this area is outside the immediately visible area on opening the start page.
Recommendation/solution idea	Responsive adaptation of the design to all screen sizes (arrangement, font size, etc.)

**Usability problem 2:** Label of ‘Contact persons’ button not clear	
Severity level	2
Location of the problem	Home page
Category	Comprehensibility
Subcategory	Button label
Description	The test person does not understand that the button label "Contact persons" refers to healthcare centers and self-help groups, but thinks that the SATURN team's contact persons can be found there.
Number of participants	1
Possible cause	The term "contact persons" can be misunderstood.
Recommendation/solution idea	Add the term "healthcare centers and self-help groups" (e.g. in brackets).

**Usability problem 3:** Complicated input of confirmed diagnoses	
Severity level	1
Location of the problem	Confirmed diagnoses
Category	User-friendliness
Subcategory	Input support
Description	The test person has to open a new window for entering each new diagnosis and then save the new diagnosis.
Number of participants	1
Possible cause	(Basic) design has it set up this way.
Recommendation/solution idea	Insert an ‘Add more diagnoses’ button in the window that opens (not a new window) to allow multiple diagnoses to be entered.

**Usability problem 4: Explanatory text for diagnosis input**	
Severity level	2
Location of the problem	Confirmed diagnoses
Category	Comprehensibility
Subcategory	Explanatory text
Description	When entering the diagnosis, two explanatory texts appear in the input field: "Enter a search term here" in the input field and "Click on the icon to edit" as text attached to the icon. The test person does not know which of these different instructions to follow.
Number of participants	1 – 5
Possible cause	The solution to enable entering and editing a single diagnosis is incorrectly selected.
Recommendation/solution idea	Select another solution option in a new framework.

**Usability problem 5:** Inappropriate icons for diagnosis input	
Severity level	2
Location of the problem	Confirmed diagnoses
Category	Comprehensibility
Subcategory	Icons
Description	When entering the confirmed diagnosis, the test person does not understand the function of the two icons next to the input field (green icon, green check mark).
Number of participants	1
Possible cause	The solution to enable entering and editing a single diagnosis is incorrectly selected.
Recommendation/solution idea	Select another solution option in a new framework.

**Usability problem 6:** Lack of confirmation when saving symptoms	
Severity level	1
Location of the problem	Symptoms
Category	Feedback
Subcategory	Saving
Description	The test person does not know whether or not the symptoms entered have been saved.
Number of participants	2
Possible cause	So far, the system returns no confirmation message.
Recommendation/solution idea	Set up the system to enable a confirmation message, ensuring system consistency.

**Usability problem 7:** Input of symptoms not already stored in system not possible	
Severity level	3
Location of the problem	Symptoms
Category	Content
Subcategory	Symptoms input
Description	The test person does not see any option to enter symptoms not listed.
Number of participants	4
Possible cause	System has not yet provided for this option.
Recommendation/solution idea	Expand and optimize input support to offer more options and allow synonyms.<br>Save new symptoms under ‘Other symptoms’.<br>(An additional field should be created to enable input of symptoms not listed under ‘Other symptoms’.)

**Usability problem 8:** Poor recognition of the symptom input field	
Severity level	2
Location of the problem	Symptoms
Category	Layout
Subcategory	Input field
Description	The test person overlooks the symptom input field.
Number of participants	1
Possible cause	The input field is not recognizable as a field; the arrow for selecting the symptoms is relatively small and positioned to the far right.
Recommendation/solution idea	Make the input field clearly recognizable as such (not just a line; input fields should have consistent design) and use a larger input arrow placed further to the left.

**Usability problem 9:** Excessive time required to input medication plan	
Severity level	2
Location of the problem	Medications
Category	User-friendliness
Subcategory	Time required
Description	The test person has to manually enter all medications, although they are already stored in the medical practice management software (PMS, or PVS in German) system. This is very time-consuming.
Number of participants	3
Possible cause	Transfer is (currently) not possible.
Recommendation/solution idea	A feasibility study needs to be done on an interface based on the standardized national medication plan.<br>Voice input must be checked.

**Usability problem 10:** Input of the dose for drug combinations unclear	
Severity level	3
Location of the problem	Medications
Category	Content
Subcategory	Medication input
Description	The test person does not automatically see how to enter the dose of both drugs for a drug combination.
Number of participants	1
Possible cause	This is due to the ontology stored in the system.
Recommendation/solution idea	Filter medication option for individual medications.<br>or<br>Enable input for each of the medications.

**Usability problem 11:** Input of medications not already stored in system not possible	
Severity level	3
Location of the problem	Medications
Category	Content
Subcategory	Medication input
Description	The test person does not see any way to enter non-listed drugs or drug variants (in this case metoprolol ext. release).
Number of participants	2
Possible cause	System has not yet provided for this option.
Recommendation/solution idea	Develop field or input strategy for ‘Other medications.<br>(An additional field should be created for entering drugs not stored in system labeled ‘Other medications’).

**Usability problem 12:** ‘Night’ medication administration time missing	
Severity level	3
Location of the problem	Medications
Category	Content
Subcategory	Medication input
Description	The test person has no option for documenting the time of medication administration as "Night".
Number of participants	1
Possible cause	System has not yet provided for this option.
Recommendation/solution idea	‘Night’ should be added to the existing 3 times as one more option.

**Usability problem 13:** Awkward prioritization of medication preselection	
Severity level	1
Location of the problem	Medications
Category	Layout
Subcategory	Structure
Description	Contrary to expectations, drug combinations are mentioned in the list before individual drugs (the case of amlodipine).
Number of participants	1
Possible cause	Amlodipine is also listed as the second drug in a combination drug (e.g. lisinopril and amlodipine) in the underlying ATC classification list.
Recommendation/solution idea	Possible new sorting of the list: first all single agents, then all combinations of 2, then all combinations of 3.<br>-> Filter and sort according to one's own criteria.

**Usability problem 14:** Non-adaptive labeling of the navigation bar	
Severity level	1
Location of the problem	Laboratory and vital signs
Category	Navigation
Subcategory	Page navigation with bar
Description	Labels of the steps in the navigation bar disappear on small screens.
Number of participants	1
Possible cause	Current implementation in the form of responsive design.
Recommendation/solution idea	Labeling that automatically adjusts font size or another support solution informing the user of which step they are at (for small screens).

**Usability problem 15:** Large number of stored units	
Severity level	2
Location of the problem	Laboratory and vital signs
Category	User-friendliness
Subcategory	Input support
Description	The test person is irritated by the large number of units saved.
Number of participants	2
Possible cause	There is a very large number of potential units (depending on the lab).
Recommendation/solution idea	Use an open-text form field with input support instead of a drop-down menu.

**Usability problem 16:** Excessive time required to input lab values	
Severity level	2
Location of the problem	Laboratory and vital signs
Category	User-friendliness
Subcategory	Time required
Description	The test person has to enter a lot of details on the lab values. This is very time-consuming.
Number of participants	1
Possible cause	A lot of details are needed to assess lab values.<br>Direct transfer from the lab results document is not possible.
Recommendation/solution idea	Transfer the values from the PMS/LVS.<br>Voice input must be checked.

**Usability problem 17:** Inconsistent pre-set options for laboratory values	
Severity level	2
Location of the problem	Laboratory and vital signs
Category	Content
Subcategory	Lab values input
Description	The two contradictory evaluations ‘abnormal’ and ‘without pathological findings’ (o.p.B. in German) can both be entered simultaneously for a lab value.
Number of participants	1
Possible cause	‘o.p.B.’ is set as a default and must be removed manually.
Recommendation/solution idea	**No default setting** for ‘o.p.B.’ ..

**Usability problem 18:** Unusual naming of stored laboratory values	
Severity level	2
Location of the problem	Laboratory and vital signs
Category	Content
Subcategory	Lab values input
Description	The test person cannot find the lab value under the expected label or abbreviation (TSH, GFR, fT4, free T4, creatinine).
Number of participants	5
Possible cause	There are no abbreviations stored in the system for the lab values and values cannot be found in the options using everyday language.
Recommendation/solution idea	Common abbreviations for lab values should also be stored so that lab values can be found by searching for abbreviations and making the search more specific.

**Usability problem 19:** Contents of ‘CBC (*kleines Blutbild*) / extended CBC (*großes Blutbild*)’ not clear	
Severity level	1
Location of the problem	Laboratory and vital signs
Category	Content
Subcategory	Lab values input
Description	The test person does not know exactly what the terms mean.
Number of participants	1
Possible cause	There is (apparently) no standard usage of the terms ‘CBC (*kleines Blutbild*) / extended CBC (*großes Blutbild*)’ across the entire medical field.
Recommendation/solution idea	Additional text should explain the lab values included or the choice of another implementation/other type of input support.

**Usability problem 20:** Imprecise labeling of blood pressure input field	
Severity level	1
Location of the problem	Laboratory and vital signs
Category	Content
Subcategory	Entering vital signs
Description	The designation selected for entering the blood pressure (value/value) is imprecise because it does not precisely define which input field is intended for the systolic and which for the diastolic blood pressure values.
Number of participants	1
Possible cause	Until now, the system has only used the term ‘value’.
Recommendation/solution idea	Change the designation to: systolic value / diastolic value.

**Usability problem 21:** Poor localization of the laboratory value input field	
Severity level	1
Location of the problem	Laboratory and vital signs
Category	Layout
Subcategory	Clarity
Description	The test person has to search a very long time to find the field for entering new lab values.
Number of participants	2
Possible cause	Since the area with the ’Lab value sets’ takes up a lot of space on the page, it is easy to overlook the ’normal’ lab value entry at the bottom.
Recommendation/solution idea	Adaptation of the lab values section for greater clarity

**Usability problem 22:** Visibility of the findings text lacking	
Severity level	1
Location of the problem	Laboratory and vital signs
Category	Layout
Subcategory	Clarity
Description	The information in the ‘Findings text’ field is not displayed on the lab values page after saving.
Number of participants	1 – 5
Possible cause	There is no column on the lab value page that displays the information entered in the ‘Findings text’ field.
Recommendation/solution idea	Add a ‘Findings text’ column to the table of saved lab values.

**Usability problem 23: Label of ‘Insert lab value set’ button incomprehensible**	
Severity level	3
Location of the problem	Laboratory and vital signs
Category	Comprehensibility
Subcategory	Button label
Description	The test person does not understand the meaning and function of ‘Insert lab value set’ .
Number of participants	5
Possible cause	The ter m ‘Insert lab value set’ is not common. It is not clear what to expect with this term.
Recommendation/solution idea	New design of the input support.

**Usability problem 24:** ‘Positive/Negative’ label not suitable for laboratory results	
Severity level	2
Location of the problem	Laboratory and vital signs
Category	Comprehensibility
Subcategory	Button label
Description	The test person does not understand the meaning of the ‘Positive/Negative’ check mark option.
Number of participants	2
Possible cause	The options ‘positive’ and ’negative’ are only suitable for some lab tests, e.g. detection of bacteria. Many lab values cannot be assigned in this way, so confusing.
Recommendation/solution idea	New design of the lab value input.

**Usability problem 25:** Input of instrumental examination findings not already stored in system not possible	
Severity level	3
Location of the problem	Examinations
Category	Content
Subcategory	Enter findings
Description	The test person does not have the option of entering instrumental examination findings (e.g. MRI).
Number of participants	1
Possible cause	Examinations can only be selected from a drop-down menu with a few options.
Recommendation/solution idea	Add open-text form field for entering findings not stored in system?

**Usability problem 26:** Unclear function of the Info icon	
Severity level	1
Location of the problem	Examinations
Category	Comprehensibility
Subcategory	Icons
Description	The test person does not know what function the info icon has.
Number of participants	1
Possible cause	The info icon looks as if you could click on it to get more information.
Recommendation/solution idea	Change the design of the info icon or remove it completely.

**Usability problem 27:** Label of 'Go to case input' button incomprehensible	
Severity level	1
Location of the problem	Results
Category	Comprehensibility
Subcategory	Button label
Description	The test person does not understand the meaning of the 'Go to case input'button.
Number of participants	1
Possible cause	It is unclear what to expect if one clicks on the 'Go to case input' button.
Recommendation/solution idea	Relabel the button, e.g. to Create & edit current case' or something similar.

**Usability problem 28:** Lack of confirmation when saving the confirmed diagnosis	
Severity level	1
Location of the problem	Case closure
Category	Feedback
Subcategory	Saving
Description	The test person cannot tell whether the confirmed diagnosis has been saved.
Number of participants	1
Possible cause	No confirmation message from the system yet.
Recommendation/solution idea	Set up the system to enable a confirmation message, ensuring system consistency.

**Usability problem 29:** Input of confirmed diagnosis only possible with ICD code	
Severity level	3
Location of the problem	Case closure
Category	Content
Subcategory	Diagnoses input
Description	The test person does not see any option of entering diagnoses not stored in system.
Number of participants	4
Possible cause	Diagnoses options to select from are limited to ICD codes. However, not every diagnosis has its own ICD code (e.g. rare diseases), so you will not find every diagnosis as an option to select.
Recommendation/solution idea	1) OrphaCodes should be added to diagnoses.<br>2) Synonyms should be added to the diagnoses.

**Usability problem 30: Incomplete display of the ICD text**	
Severity level	1
Location of the problem	Case closure
Category	Content
Subcategory	ICD code display
Description	Only part of the ICD text is visible when the case is closed.
Number of participants	1
Possible cause	No space to display the full text of the ICD code in the drop-down menu
Recommendation/solution idea	Fine-tune the design so that the complete text is visible.

**Usability problem 31: Lack of instructions for input**	
Severity level	3
Location of the problem	Case closure
Category	Comprehensibility
Subcategory	Input field
Description	The test person does not understand what should be entered on the ‘Close case’ page.
Number of participants	4
Possible cause	The page has no heading.
Recommendation/solution idea	Add a heading for this page or instruction, e.g. ’Enter the confirmed diagnosis’.

**Usability problem 32:** Unclear function of the ‘Diagnostic history’ input field	
Severity level	1
Location of the problem	Case closure
Category	Comprehensibility
Subcategory	Input field
Description	The test person does not understand what to enter in the ‘Diagnostic history’ field.
Number of participants	1
Possible cause	The term ‘diagnostic history’ is not commonly used.
Recommendation/solution idea	Rename the text field to ’Additional notes’ or ’Notes on the history of the diagnosis’.

**Usability problem 33:** Direct navigation back to home page not visible	
Severity level	2
Location of the problem	Patient overview
Category	Navigation
Subcategory	Page navigation without any bar
Description	The test person cannot tell how to return to the start page.
Number of participants	2
Possible cause	There is no button specifically labeled as ‘Start page’.
Recommendation/solution idea	On this page, add a button ‘Back to start page’ and add the term ’Start page’ as text or note to the SATURN symbol at the top left.

**Usability problem 34: Closed cases marking is lacking**	
Severity level	3
Location of the problem	Patient overview
Category	Feedback
Subcategory	Feedback lacking
Description	The test subjects cannot tell whether or not a case has already been closed.
Number of participants	2
Possible cause	In the patient overview, the ‘Completed’ cases are not marked as ‘Completed’.
Recommendation/solution idea	For closed cases, add a ‘Closed’ marker and gray out the closed cases.

**Usability problem 35: Lack of export/print function**	
Severity level	1
Location of the problem	Contact persons
Category	User-friendliness
Subcategory	Operation
Description	The test person has no option to print or export information on contact persons.
Number of participants	1
Possible cause	No print/export function available.
Recommendation/solution idea	Add print or export function.

**Usability problem 36: Lack of filter functions**	
Severity level	1
Location of the problem	Contact persons
Category	User-friendliness
Subcategory	Operation
Description	The test person has no option to filter the list of contact persons according to the search criteria of location and adults/children.
Number of participants	1
Possible cause	There is no corresponding filter function on the ‘Contact persons’ page.
Recommendation/solution idea	Add a corresponding filter function/search function.

**Usability problem 37:** Poor localization of the results list of patient organizations	
Severity level	2
Location of the problem	Contact persons
Category	Layout
Subcategory	Clarity
Description	(On the ‘Contact persons’ page, you have to scroll all the way down to find the list of patient organizations below the clinics).<br><br>The test person overlooks the results list of the patient organizations.
Number of participants	2
Possible cause	It is not clear enough at the top of the page that the item ‘Patient organizations’ is to be expected further down.
Recommendation/solution idea	Optimize the page design so that users can see both at the top and can ‘jump’ to the corresponding table.

**Usability problem 38:** Label of ‘Back to overview’ button imprecise	
Severity level	1
Location of the problem	Contact persons
Category	Comprehensibility
Subcategory	Button label
Description	The test person cannot tell that they are returning to the start page with the ‘Back to overview’ button .
Number of participants	1
Possible cause	The term ‘Overview’ is imprecise.
Recommendation/solution idea	Rename the ‘Back to overview’ button to ‘Back to start page’.

**Idea 1: Lack of symptom duration**	
Severity level	0
Location of the problem	Symptoms
Category	Content
Subcategory	Symptoms input
Description	The duration of symptoms is not queried.
Number of participants	2
Possible cause	System has not yet provided for this option.
Recommendation/solution idea	Develop a field or input strategy for the ‘duration’ of the symptoms.

**Idea 2: Lack of symptom weighting**	
Severity level	0
Location of the problem	Symptoms
Category	Content
Subcategory	Symptoms input
Description	The symptom weighting is not queried.
Number of participants	1
Possible cause	System has not yet provided for this option.
Recommendation/solution idea	Develop a field or input strategy for ‘weighting’ the symptoms.

**Idea 3: Lack of an option to choose rhythmic/arrhythmic for blood pressure**	
Severity level	0
Location of the problem	Laboratory and vital signs
Category	Content
Subcategory	Entering vital signs
Description	The test person has no input option for input of rhythmic/arrhythmic.
Number of participants	1
Possible cause	Not provided in the version to date.
Recommendation/solution idea	Add the (checkable) options ‘Rhythmic’ and ’Arrhythmic”’by heart rate entry.

**Idea 4: Lack of physical examination findings input**	
Severity level	0
Location of the problem	Examinations
Category	Content
Subcategory	Examinations input
Description	The test person has no option to enter physical examination findings (e.g. enlarged liver, enlarged spleen).
Number of participants	1
Possible cause	Only instrumental examinations are stored on the ‘Examinations’ page. There is no page for ‘physical examination findings’.<br><br>Not previously provided for in the system.
Recommendation/solution idea	Find a good solution for entering findings across all categories.<br>Add one, if it can be used by the system.

**Idea 5: Lack of marking function**	
Severity level	0
Location of the problem	Results
Category	User-friendliness
Subcategory	Operation
Description	The test person cannot mark any content in the results list, e.g. to gray out diagnoses that have already been excluded.
Number of participants	1
Possible cause	There is no such functionality yet built into the system.
Recommendation/solution idea	
